# *In vitro* and *in vivo* Effects of Free and Chalcones-Loaded Nanoemulsions: Insights and Challenges in Targeted Cancer Chemotherapies

**DOI:** 10.3390/ijerph111010016

**Published:** 2014-09-26

**Authors:** Evelyn Winter, Carine Dal Pizzol, Claudriana Locatelli, Adny H. Silva, Aline Conte, Louise D. Chiaradia-Delatorre, Ricardo J. Nunes, Rosendo A. Yunes, Tânia B. Creckzynski-Pasa

**Affiliations:** 1 Department of Pharmaceutical Sciences, Federal University of Santa Catarina, P.O. Box 476, Florianópolis, SC 88040-900, Brazil; E-Mails: eve_winter@hotmail.com (E.W.) carinedp@gmail.com (C.D.P.); adnyh@yahoo.com.br (A.H.S.); 2 Department of Pharmacy, University of West of Santa Catarina, Videira, SC 89560-000, Brazil; E-Mails: claudrilocatelli@gmail.com (C.L.); alineconte91@gmail.com (A.C.); 3 Department of Chemistry, Federal University of Santa Catarina, P.O. Box 476, Florianópolis, SC 88040-900, Brazil; E-Mails: louisedc@gmail.com (L.D.C.-D.); nunesricardoj@gmail.com (R.J.N.); ryunes@msn.com (R.A.Y.)

**Keywords:** nanomedicine, nanoemulsion, chalcones, leukemia, chemotherapy

## Abstract

Several obstacles are encountered in conventional chemotherapy, such as drug toxicity and poor stability. Nanotechnology is envisioned as a strategy to overcome these effects and to improve anticancer therapy. Nanoemulsions comprise submicron emulsions composed of biocompatible lipids, and present a large surface area revealing interesting physical properties. Chalcones are flavonoid precursors, and have been studied as cytotoxic drugs for leukemia cells that induce cell death by different apoptosis pathways. In this study, we encapsulated chalcones in a nanoemulsion and compared their effect with the respective free compounds in leukemia and in non-tumoral cell lines, as well as in an *in vivo* model. Free and loaded-nanoemulsion chalcones induced a similar anti-leukemic effect. Free chalcones induced higher toxicity in VERO cells than chalcones-loaded nanoemulsions. Similar results were observed *in vivo*. Free chalcones induced a reduction in weight gain and liver injuries, evidenced by oxidative stress, as well as an inflammatory response. Considering the high toxicity and the side effects induced generally by all cancer chemotherapies, nanotechnology provides some options for improving patients’ life quality and/or increasing survival rates.

## 1. Introduction

Cancer is among the leading causes of death worldwide, and acute lymphoblastic leukemia (ALL) is the most common form of pediatric leukemia. Although the five-year survival rate for ALL patients has increased from 83.7% in 1990 to 90.4% in 2005, leukemia is still the leading cause of cancer-related death in children [1]. Childhood ALL as well as other tumors are treated with multiple-agent chemotherapy using drugs such as anthracyclines, corticoids and methotrexate, which can result in serious acute and late complications for patients due to the toxicity in non-tumoral cells. The use of anthracyclines has been related to a high risk of cardiac failure in ALL patients [2,3,4]. In addition, high doses of corticoids and methotrexate are related to a risk of low bone mineral density in ALL-treated patients [5,6]. 

Another problem involved in leukemia is the infiltration of leukemic cells in the central nervous system (CNS) that occurs in about 6% of the patients. As conventional drugs cannot cross the blood brain barrier, intrathecal drugs administration and/or cranial irradiation are used in prophylaxis and/or treatment of patients, which might result in a high risk of secondary tumor development [7,8]. 

An important strategy to overcome the side effects of chemotherapy, and to improve the efficiency of the drugs, is to encapsulate them in nanostructured systems. Nanotechnology is a scientific field that involves the application of materials on a nanometer scale in different areas including engineering, chemistry, biology, and medicine [9]. In chemotherapy, nanotechnology might be used to increase the selectivity, stability and solubility of drugs, as well as to increase the permeability of drugs in solid tumors by the Enhanced Permeability and Retention (EPR) effect, including the blood brain barrier, which is an extramedular site highly involved in leukemia metastasis [10,11,12].

In this context, nanoemulsions have been used as biocompatible systems, in which the oil phase is dispersed as droplets in an aqueous phase and stabilized by surfactants. Alprostadil palmitate, amphotericin B, dexamethasone, flurbiprofen and vitamins are examples of therapeutic compounds that have been formulated in nanoemulsions for clinical application [13,14].

In this work we used as therapeutic strategy the encapsulation of chalcones in nanoemulsion systems. Chalcones are essential intermediate compounds in flavonoid biosynthesis in plants, and their antileukemic activity has already been detailed. The three chalcones studied here (R7, R13 and R15) have been shown to induce apoptosis in leukemic cells by different pathways at concentrations in the micromolar range [15,16]. In this study, we encapsulated each compound in nanoemulsions to compare the antileukemic activity *in vitro,* and the toxicity *in vivo* of free and chalcones-loaded nanoemulsion.

## 2. Experimental Section 

### 2.1. Materials

**Figure 1:**
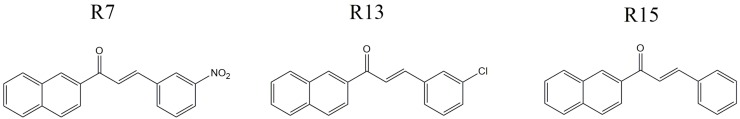
Chalcone structures.

For the manufacture of nanoparticles, Miglyol 812 liquid oil was purchased from Caelo GmbH (Hilden, Germany), lecithin S75 (Lipoid S75) was purchased from Lipoid (Steinhausen, Switzerland), and polysorbate 80 was purchased from Synth (São Paulo, Brazil). The cell culture media and fetal bovine serum were purchased from Cultilab (São Paulo, Brazil). The antibiotics penicillin/streptomycin and 2’,7’-dichlorofluorescein diacetate (DCFH-DA) were purchased from Life Technologies (Carlsbad, US). Dimethyl sulfoxide (DMSO) was purchased from Merck (Darmstadt, Germany), and all other reagents were purchased from Sigma-Aldrich (St. Louis, MO, US). Chalcones were synthesized by a research group at the Chemistry Department of Federal University of Santa Catarina (Figure 1) [17].

### 2.2. Nanoparticles Characterization

#### 2.2.1. Nanoemulsion Preparation

The formulations were obtained by an ultrasound method [18]. Briefly, the matrix lipid, surfactant lecithin S75 and chalcones were heated to 56–70 °C. After that, the aqueous solution containing PBS buffer and polysorbate 80, previously heated at the same temperature of the lipid phase, was mixed in the oil phase under stirring. Next, the sonication probe (6 mm diameter) of an ultrasonic processor (Sonics, Newtown, CT, US) was placed in the pre-emulsion and set to produce an output power with 70% amplitude for 3 min at 4 °C, leading to droplet breakage by acoustic cavitation and subsequent nanoemulsion formation. The formulations consisted of 0.5 mg/mL chalcones and 20 mg/mL lipid stabilized by 10 mg/mL of surfactant mixture. Chalcones were not added in blank nanoemulsions.

#### 2.2.2. Characterization Nanoemulsion Physicochemical Properties 

The particle size, polydispersity index (PDI) and electrophoretic mobility (zeta potential) of the nanoemulsions were measured by dynamic light scattering (DLS) in a Zetasizer Nano ZS (Malvern Instruments, Malvern, UK), equipped with 173° scattering angle. The measurements were made at 25 °C after appropriated sample dilution in ultrapure water (Merck-Millipore, Darmstad, Germany). To measure zeta potential, samples of the nanoparticle formulations were placed in a specific cell in which a potential of 150 mV was established. The zeta potential values were calculated by means of electrophoretic mobility values using Smoluchowski’s equation [19].

#### 2.2.3. High-Performance Liquid Chromatographic Analysis

The HPLC apparatus consisted of a chromatograph (PerkinElmer, Santa Clara, CA, US) equipped with a series 200 auto sampler, Series 200 binary pump, Series 200 UV-Vis detector and Series 200 vacuum degasser. The stationary and mobile phases used were a C18 Zorbax ODS (150 mm × 4.6 mm i.d., 5 μm particle size, Agilent Technologies (Santa Clara, CA, US) and methanol-water (85:15 v/v), respectively. The mobile phase was pumped in isocratic flow for 10 min. Detection was at 274 nm. The method was validated to assay the chalcones, according to the ICH guidelines [20]. 

#### 2.2.4. Determination of Encapsulation Efficiency

The amounts of chalcones encapsulated in the nanoemulsions were assayed by the HPLC method as previously described. A sample of each formulation was diluted in methanol, and the amounts of encapsulated molecules were determined by measuring the non-incorporated chalcones present in the aqueous phase after ultrafiltration/centrifugation [21] using Microcon® centrifugal filter devices (100,000 NMWL; Millipore). The chalcone encapsulation efficiencies were calculated from the difference between the concentration of the total and that of the free compounds.

#### 2.2.5. Differential Scanning Calorimetry

Thermal analysis was performed to verify the crystallinity and the incorporation of chalcones into the lipid matrix of the nanoemulsion. Differential scanning calorimetry (DSC) analyses were performed by a DSC-50 instrument (Shimadzu, Kyoto, Japan). Samples of free chalcones and chalcones-loaded nanoemulsion were weighed (2–5 mg), loaded in aluminum pans and hermetically sealed. An empty standard aluminum pan was used as reference. Scans were recorded at a heating rate of 10 °C/min from 25 °C to 200 °C. 

### 2.3. In vitro Studies

#### 2.3.1. Cell Culture

Monkey kidney epithelial cells (VERO) and acute lymphoblastic leukemia cells (L1210) were obtained from American Type Culture Cell (ATCC). VERO cells were cultured in DMEM and L1210 cells were cultured in RPMI, both supplemented with 10% heat-inactivated fetal bovine serum, 100 U/mL penicillin, 100 µg/mL streptomycin and 10 mM HEPES. The cell culture was maintained at 37 °C in a 5% CO_2_ humidified atmosphere and pH 7.4. In all experiments, viable cells were checked at the beginning of the experiment by Trypan Blue exclusion. 

#### 2.3.2. Viability Assay

The cytotoxicity of free and chalcones-loaded nanoemulsions were evaluated by 3-(4,5-dimethylthiazol-2-yl)-2,5-diphenyltetrazolium bromide (MTT) assay [22]. Vero cells (1 × 10^4^/well) and L1210 cells (5 × 10^4^/well) were seeded in 96-well culture plates. After overnight incubation, cells were exposed to free and encapsulated chalcones for 24 h at concentrations ranging from 0 to 100 µM. After 24 h, 100 µL of fresh culture medium with 10 μL of MTT (final concentration 5 mg/mL) was added and incubated for 2 h. The precipitated formazan formed was dissolved in DMSO and the absorbance was measured at 540 nm using a micro-well system reader. The results were expressed as area under the curve (AUC) calculated using GraphPad Prism5. The AUC was used to compare the cytotoxic effect among the compounds because some treatments did not achieve 50% of cell death. The selectivity index (SI) was calculated dividing the value of AUC obtained with non-tumoral cells by the value obtained with leukemic cells. 

### 2.4. In vivo Studies

#### 2.4.1. Drug Treatment and Animals

Male Swiss albino mice (6–8 weeks old) were maintained at 23 ± 2 °C with relative humidity of 50%–60% under a 12:12 h light-dark cycle with food and water *ad libitum*. Prior to performing the experimental procedures, mice were matched for body weight (30–35 g). Animals used in this study were handled in accordance with The Use on the Principles of Animal Care, previously approved by the Ethics Committee for Animal from University of Oeste de Santa Catarina (Opinion number 01/2012). Animals were divided into groups (*n* = 5 each): control, which received only the vehicle (PBS or DMSO); NE, which received the blank nanoparticle; treated groups, which received 5 mg/Kg of free or nanoemulsion-loaded chalcones. As there are currently no guidelines or standard methodologies for *in vivo* toxicity, we chose to work with the doses suggested by The Organization for Economic Co-operation and Development (OECD) guidelines 420 for investigating the oral toxicity of any new substance. The doses suggested in OECD TG 420 for investigating the toxicity of test chemicals are from 5 to 5000 mg/kg. We chose the concentration of 5 mg/Kg based on the maximum volume possible to administer to the animal by intraperitoneal pathway (10 mL/Kg) [23] and the maximum capacity of encapsulation of the chalcones into the nanoemulsion (0.5 mg/mL). A single dose was chosen to minimize the use of animals. Compound and vehicle were administered by intraperitoneal pathway injection daily for 14 days, characterizing a repeated-dose study. On the final day of the treatment, the animals were killed, blood was collected and selected organs (heart, liver, spleen, brain, stomach, lung and kidney) were removed and weighed. Tissues from brain, liver, spleen, lung, and kidney were fixed in 4% PBS-formaldehyde and processed for histopathology. Blood was used to evaluate hepatic, renal, and hematologic toxicity. The oxidative stress was assayed in the liver homogenates. Body weight was evaluated every two days and at the end of treatment.

### 2.5. Evaluation of Oxidative Stress in Liver

#### 2.5.1. Preparation of Homogenates

Livers were rapidly removed and homogenized (liver 1:10 w/v) in a buffer containing 1% Triton X-100, 150 mM NaCl, 20 mM sodium phosphate, pH 7.4. The livers were homogenized in a tissue homogenizer for 30 s on ice, followed by centrifugation at 10,000 × g for 10 min. Protein content was determined by Lowry’s method [24]. Lipid peroxidation, reactive species generation and thiols content analysis were performed with freshly prepared samples. To measure the activity of catalase, glutathione peroxidase and glutathione reductase, the supernatants were stored at −80 °C until utilization.

#### 2.5.2. ROS Determination

Intracellular free radical formation was determined using 2’,7’-dichlorofluorescein diacetate (DCFH-DA), which is oxidized to dichlorofluorescein (DCF) in the presence of ROS [25]. Liver homogenates (200 µg) were incubated with 0.1 µM DCFH-DA in 96-well plates for 30 min at 37 °C. The DCF fluorescence signal was measured using a Perkin–Elmer LS55 spectrofluorimeter. 

#### 2.5.3. Enzyme Assays

Glutathione peroxidase (GPx) was assayed using 200 µg of protein, and NADPH oxidation was monitored spectrophotometrically at 340 nm [26]. Catalase activity was determined using 100 µg of protein [27]. In this assay, the disappearance of H_2_O_2_ was evaluated by measuring the decrease in absorbance at 240 nm. Glutathione reductase (GR) was assayed using 200 µg of protein and the NADPH oxidation, which resulted from the reduction of oxidized glutathione (GSSG) by GR, determined spectrophotometrically at 340 nm [28]. Glutathione S-transferase (GST) was assayed using 100 µg of protein. This assay is based on the conjugation of reduced glutathione (GSH) with 1-chloro-2,4-dinitrobenzene (CDNB) by GST. The conjugate was detected spectrophotometrically at 340 nm [29]. Results were calculated as µmoL/min/µg of protein and expressed as percentage considering the control values as 100%. Controls values: 12 µmoL/min/µg protein for catalase; 1 µmoL/min/µg protein for GR; 14 µmoL/min/µg protein for GPx and GST.

#### 2.5.4. Lipid Peroxidation Measurements

Thiobarbituric acid reactive species (TBARS) levels were determined according to the method already established, in which malondialdehyde—the major end product of fatty acid peroxidation—reacts with thiobarbituric acid to form a colored complex [30]. Freshly prepared tissue homogenates were sequentially mixed in test tubes with equal volumes of 60 mM Tris-HCl, pH 7.4, 0.1 mM DPTA buffer, 12% TCA and 0.73% thiobarbituric acid (TBA), stirring after each addition. The tubes were kept at 100 °C for 1 h and subsequently cooled and centrifuged (10,000 × g for 5 min). The absorbance of the supernatant was measured at 535 nm. The TBARS concentration in the samples was calculated using an analytical curve of malondialdehyde in nmoL/mg protein, and the results were expressed as percentage considering the control value as 100%. Control value: 21 nmoL/mg protein.

#### 2.5.5. Non-Protein Thiol Determination

GSH concentration in the samples was determined by the 5,5-dithiobis-2-nitrobenzoic acid (DTNB) method [31]. This method is based on the reaction of GSH with DTNB, generating a thiolate anion (TNB), whose yellow color is measured spectrophotometrically at 412 nm. To determine the GSH content the samples were added to a reaction medium containing 20 µM DTNB in 200 mM sodium phosphate buffer, pH 8.0. The formation of TNB was monitored after 10 min at 405 nm. The GSH concentration was calculated using an analytical curve of GSH in µmoL/mg protein, and the results were expressed as percentage considering the control value as 100%. Control value: 12 µmoL/mg protein.

### 2.6. Biochemical Parameters 

To evaluate the hepatic function the blood samples were centrifuged at 200 × g for 10 min at room temperature, the serum was separated to measure aspartate transaminase (AST) and alanine transaminase (ALT) activities, as well as the content of total protein and albumin. Renal function was evaluated based on serum urea and creatinine levels. The determination of the content of plasma cholesterol and triglycerides was also performed to verify if the lipid nanoparticles could affect the normal lipid levels. Commercially available kits (Labtest Diagnóstica SA, Lagoa Santa, Brazil) were used for the biochemical analysis.

### 2.7. Hematological Evaluation

Hematological parameters such as red blood cell number (RBC), white blood cell number (WBC), lymphocyte and neutrophil counts were evaluated according to the method described elsewhere [32]. The serum content of hemoglobin, hematocrit, mean corpuscular hemoglobin (MCH), mean corpuscular volume (MCV) and mean corpuscular hemoglobin concentration (MCHC) were evaluated according to the method described elsewhere [33].

### 2.8. Histopathological Analysis

Tissues recovered from the necropsy were fixed in 10% formalin, embedded in paraffin, sectioned, and stained with hematoxylin and eosin (HE) for histological examination using standard techniques. After hematoxylin/eosin staining, the slides were observed and photos were taken using an optical microscope (BH2-RFCA, Olympus, Hamburg, Germany). All the identity and analysis of the pathology slides were blind to the pathologist. Tissue sections were observed under a microscope at 400× magnification. 

### 2.9. Statistical Analysis

The results were presented as means ± standard error of mean (SE). Statistical significance was assessed by ANOVA followed by Dunnett’s test, and a *p* value less than 0.05 was considered significant.

## 3. Results

### 3.1. Nanoparticles Characterization

The functional performance of nanoparticle-based delivery systems depends on the physicochemical properties of the nanoparticles, such as size, morphology, charge, and physical state [34,35]. Values of the particle size, polydispersity index (PDI), zeta potential (ZP) and encapsulation efficiency of the developed nanoemulsions are shown in Table 1. After chalcone encapsulation, the mean diameters of the particles did not change. The formulations presented a negative surface charge (zeta potential) as expected for the types of mixture of surfactant used, which allows a strong electrostatic repulsion that prevents particle aggregation. The PDI values indicate that the free and loaded nanoparticles obtained by the displacement solvent method were homogeneous, and that the method employed was reproducible and stable. The encapsulation efficiency (EE) values were higher than 86% for formulations with chalcones. Therefore, the high values of EE observed for different chalcones can be explained by the higher affinity of the molecules to nanoparticles than to the aqueous solution phase during the preparation process.

**Table 1:** Nanoemulsion characterization.

	Mean Diameter (nm)	PDI	ZP (mV)	EE (%)
NE/R7	124 ± 6	0.24 ± 0.01	−24 ± 5.1	86
NE/R13	107 ± 2	0.19 ± 0.01	−17 ± 2.8	93
NE/R15	110 ± 3	0.17 ± 0.01	−19 ± 4.2	93
NE	117 ± 4	0.19 ± 0.02	−22 ± 1.4	-

PDI–polydispersity index; ZP–zeta potential; EE–encapsulation efficiency; NE–chalcones−free nanoemulsion.

#### 3.1.1. Differential Scanning Calorimetry Investigation

Differential scanning calorimetry (DSC) provided information on the structural organization of the bioactive molecules and lipid matrix within the nanoparticles. DSC allows the evaluation of lipid-drug interactions, even if the drug and the lipid matrix are molecularly dispersed. The DSC curves of free chalcones (R7, R13 and R15) showed one endothermic transition at 140 °C, 121 °C and 101 °C respectively, corresponding to melting of chalcones. For chalcone-free nanoemulsion the DSC curves did not demonstrate any endothermic peak, because the lipid matrix used is an oil with no melting point. For chalcone-loaded nanoemulsions, the DSC curves showed no thermal transitions, indicating that the molecules were successfully entrapped in the nanoemulsion and are dispersed in a non-crystalline state within the lipid matrix (Figure S1—Supplementary Material).

### 3.2. In vitro Cell Toxicity

To determine the antitumoral and the toxic effects of free and encapsulated chalcones, a leukemic cell (L1210) and a non-tumoral cell (VERO) were used, respectively. The effects of chalcones are presented in Table 2. Control cells treated only with vehicle (PBS or DMSO) were considered with an area under the curve (AUC) value of 10,000. The blank nanoemulsion (NE) seems to be non-toxic (AUC~10,000), indicating that this formulation is suitable for use. Free chalcones induced a high toxicity in leukemic cells, and this effect was only slightly lower in non-tumoral cells as the selectivity index (~1) indicates. The encapsulation of chalcones decreased the toxicity of R7 and R15 in non-tumoral cells (~2-fold), inducing an increase in the selectivity index. R13-loaded nanoemulsion did not induce toxicity in L1210 and VERO cells. Chalcone R13 presented the highest hydrophobicity as evidenced by the partition coefficient (5.14) in comparison with the other chalcones (R7: 4.43 and R15: 4.58). The loss of R13 activity in the nanostructured form could be related to the high affinity of the chalcone for the lipid matrix, resulting in a non-release of the drug from the matrix.

**Table 2:** Cytotoxicity of free and chalcones-loaded nanoemulsion in leukemic and non-tumoral cells.

AUC—24 h Incubation			
	Leukemic Cell	Non-Tumoral Cell	
	L1210	VERO	SI
Control (PBS)	10,000	10,000	-
NE	8778 ± 52	9351 ± 297	-
R7	3344 ± 57	4180 ± 7	1.2
NE/R7	4829 ± 43	7684 ± 223	1.6
R13	3542 ± 190	5064 ± 327	1.4
NE/R13	8562 ± 517	8624 ± 487	1.0
R15	3509 ± 181	3996 ± 328	1.1
NE/R15	4942 ± 35	7300 ± 524	1.5

AUC—area under the curve; SI—selectivity index.

### 3.3. In vivo Toxicity

Based on *in vitro* results, which demonstrated a lower toxicity of chalcones-loaded nanoemulsion in relation to free chalcones in non-tumoral cells, *in vivo* studies were performed. During the 14 days of treatment, the animals were weighed every two days and the results are shown in the Table 3 and Table 4. For the statistical analysis, the weight of each day was compared with the weight of the first day (day 0) within the same group. Already by day 4, the non-treated animals presented a significant increase in body weight, while the animals treated with free chalcones did not present a significant increase until the last day of treatment. These results could be related to a toxic effect of chalcones (Table 3). Animals treated with chalcones-loaded nanoemulsion presented a similar or larger increase in body weight than the animals of the control group. This result indicates that the encapsulation avoided the toxic effect of the free compounds (Table 4). 

**Table 3:** Evaluation of animals’ weight during the free chalcone treatment.

Days	Control (g)	R7 (g)	R13 (g)	R15 (g)
0	31.0 ± 1.1	30.8 ± 1.1	28.8 ± 0.8	31.0 ± 0.8
2	31.3 ± 0.9	31.8 ± 1.4	30.0 ± 0.6	32.7 ± 0.6
4	32.3 ± 0.9 ^*^	32.0 ± 1.1	29.5 ± 0.6	32.5 ± 0.5
6	32.7 ± 1.2 ^**^	32.1 ± 1.1	29.5 ± 1.2	32.7 ± 0.3
8	32.3 ± 0.9 ^*^	32.3 ± 1.4	29.5 ± 0.9	32.7 ± 0.7
10	32.7 ± 1.2 ^**^	31.8 ± 1.4	29.7 ± 0.8	32.8 ± 0.7
12	33.3 ± 0.9 ^***^	31.8 ± 1.3	29.8 ± 0.5	32.7 ± 0.6
14	33.0 ± 1.1 ^**^	31.7 ± 1.6	29.5 ± 0.7	32.3 ± 0.9
Variation	~2 g	~0.9	~0.7	~1.3

* *p* ≤ 0.05; ** *p* < 0.01; *** *p* < 0.001 in relation to the control using repeated−measures ANOVA followed by Dunnet’s test when compared with day 0 within the same group. Variation was calculated by subtracting the weight on day 14 from the weight on day 0.

**Table 4:** Evaluation of animals’ weight during the chalcone-loaded nanoemulsion treatment.

Days	Control (g)	NE (g)	NE/R7 (g)	NE/R13 (g)	NE/15 (g)
0	29.0 ± 2.1	29.2 ± 3.2	28.2 ± 1.3	28.3 ± 1.2	28.2 ± 1.6
2	29.7 ± 2.3	29.0 ± 3.2	28.7 ± 1.4	28.8 ± 1.3	29.3 ± 1.4
4	30.7 ± 2.3	29.4 ± 2.5	29.7 ± 1.4	29.3 ± 1.2	29.3 ± 1.5
6	29.0 ± 1.5	30.2 ± 2.5	28.7 ± 1.0	29.5 ± 1.1	29.7 ± 1.5
8	30.7 ± 2.3	30.4 ± 2.2	29.2 ± 1.5	29.2 ± 0.9	30.2 ± 1.5
10	30.7 ± 2.3	30.6 ± 2.3	29.5 ± 1.3	31.2 ± 0.8 ^***^	30.8 ± 1.8 ^*^
12	31.3 ± 1.7 ^*^	32.2 ± 2.2 ^**^	29.8 ± 1.2 ^*^	31.5 ± 1.2 ^***^	30.3 ± 1.0
14	32.7 ± 1.3 ^***^	32.2 ± 2.1 ^*^	31.5 ± 0.8 ^***^	33.8 ± 1.0 ^***^	32.8 ± 1.8 ^***^
Variation	~3.7	~2.2	~3.3	~5.5	~4.6

* *p* ≤ 0.05; ** *p* < 0.01; *** *p* < 0.001 in relation to the control using repeated-measures ANOVA followed by Dunnet’s test when compared with day 0 within the same group. Variation was calculated by subtracting the weight on day 14 from the weight on day 0.

To improve the toxicity analysis, some important organs from the animals were also weighed at the end of the treatment and the results are shown in the Table 5 and Table 6. Animals treated with free chalcones did not present alterations in organ weight. An enlargement of the livers of animals treated with all chalcones-loaded nanoemulsion and with blank nanoemulsion (NE) was observed. This increase could be the result of lipid deposition in the organ. Conversely, NE did not induce significant alterations in other biochemical or histological parameters, indicating that the increase in liver weight observed is not related to toxicity. 

**Table 5:** Evaluation of organs weight after the 14 days of free chalcones treatment.

Parameters	Control (g)	R7 (g)	R13 (g)	R15 (g)
Spleen	0.18 ± 0.03	0.23 ± 0.03	0.21 ± 0.01	0.27 ± 0.03
Liver	2.21 ± 0.14	2.15 ± 0.09	2.02 ± 0.03	2.46 ± 0.16
Heart	0.23 ± 0.02	0.21 ± 0.01	0.20 ± 0.04	0.23 ± 0.01
Lung	0.25 ± 0.01	0.28 ± 0.01	0.26 ± 0.01	0.29 ± 0.01
Brain	0.42 ± 0.04	0.35 ± 0.02	0.36 ± 0.01	0.39 ± 0.01
Kidneys	0.65 ± 0.03	0.70 ± 0.06	0.62 ± 0.01	0.69 ± 0.03
Stomach	0.24 ± 0.03	0.30 ± 0.02	0.30 ± 0.01	0.31 ± 0.04

**Table 6:** Evaluation of organs weight after the 14 days of chalcones-loaded nanoemulsion treatment.

Parameters	Control (g)	NE (g)	NE/R7 (g)	NE/R13 (g)	NE/15 (g)
Spleen	0.17 ± 0.01	0.20 ± 0.02	0.25 ± 0.03	0.24 ± 0.01	0.20 ± 0.01
Liver	1.58 ± 0.13	2.15 ± 0.09	1.96 ± 0.06	2.05 ± 0.08	1.96 ± 0.14
Heart	0.18 ± 0.01	0.17 ± 0.02	0.19 ± 0.01	0.20 ± 0.06	0.19 ± 0.01
Lung	0.21 ± 0.01	0.20 ±0.01	0.25 ± 0.01	0.25 ± 0.01	0.25 ± 0.02
Brain	0.39 ± 0.04	0.32 ± 0.02	0.38 ± 0.03	0.37 ± 0.01	0.38 ± 0.02
Kidneys	0.39 ± 0.04	0.23 ±0.02	0.48 ± 0.03	0.50 ± 0.03	0.48 ± 0.04
Stomach	0.33 ± 0.04	0.24 ± 0.02	0.26 ± 0.02	0.33 ± 0.02	0.29 ± 0.02

### 3.4. Biochemical Parameter Investigation

The results of biochemical studies are summarized in Table 7 and Table 8. No biochemical parameter alterations of clinical relevance were observed. An increase of triglycerides was observed in free chalcone-treated animals, while the encapsulation of R13 and R15 avoided these effects. Chalcones also induced a decrease in total cholesterol that could be related to a hypolipidemic effect of chalcones, as demonstrated in another study [36]. 

**Table 7:** Biochemical parameters analysis after 14 days of treatment with free chalcones.

Parameters	Control	R7	R13	R15
ALT (UI/L)	159 ± 12	101 ± 4	109 ± 4	111 ± 5
AST (UI/L)	161 ± 19	161 ± 4	171 ± 4	170 ± 3
Albumin (g/dL)	2.2 ± 0.1	2.4 ± 0.1	2.4 ± 0.1	2.3 ± 0.1
Total Proteins (g/dL)	5.8 ± 0.2	6.0 ± 0.2	5.2 ± 0.1	5.5 ± 0.1
Urea (mg/dL)	62 ± 4	78 ± 4	74 ± 2	69 ± 6
Creatinine (mg/dL)	0.5 ± 0.04	0.5 ± 0.01	0.5 ± 0.01	0.5 ± 0.02
Triglycerides (mg/dL)	75 ± 4	129 ± 11 ^**^	125 ± 9 ^*^	115 ± 11 ^*^
Total Cholesterol (mg/dL)	118 ± 9	77 ± 3 ^**^	85 ± 3 ^**^	78 ± 4 ^**^

^*^ *p* ≤ 0.05; ^**^ *p* < 0.01 using ANOVA followed by Dunnet’s test when compared with the control group.

**Table 8:** Biochemical parameters analysis after 14 days of treatment with chalcone-loaded nanoemulsions.

Parameters	Control	NE	NE/R7	NE/R13	NE/15
ALT (UI/L)	159 ± 12	157 ± 10	104 ± 4	111 ± 4	100 ± 2
AST (UI/L)	161 ± 19	145 ± 6	168 ± 2.7	165 ± 8	178 ± 6
Albumin (g/dL)	2.2 ± 0.1	2.0 ± 0.1	3.4 ± 0.1	2.7 ± 0.2	2.3 ± 0.1
Total Proteins (g/dL)	5.8 ± 0.2	5.9 ± 0.2	5.9 ± 0.3	5.6 ± 0.3	5.6 ± 0.1
Urea (mg/dL)	62 ± 4	61 ± 5	74 ± 7.5	64 ± 3.6	50 ± 2.2
Creatinine (mg/dL)	0.5 ± 0.04	0.4 ± 0.01	0.4 ± 0.02	0.5 ± 0.03	0.5 ± 0.05
Triglycerides (mg/dL)	75 ± 4	96 ± 14	145 ± 9 ^**^	99 ± 9	87 ± 10
Total Cholesterol (mg/dL)	118 ± 9	120 ± 6	94 ± 6	92 ± 2 ^*^	87 ± 7 ^*^

* *p* ≤ 0.05; ** *p* < 0.01 using ANOVA followed by Dunnet’s test when compared with the control group.

### 3.5. Hematological Evaluation

Table 9 and Table 10 show the results of hematological parameters evaluated after the treatment with free and chalcone-loaded nanoemulsion. A slight increase in neutrophil cells was induced by free chalcone R15, indicating an early inflammatory process. This increase was avoided when the compound was encapsulated. The increase of leucocytes, neutrophils and mononuclear cells induced by blank nanoemulsion (NE), as was not the case with chalcones themselves, could be the result of immune system stimulation by the nanoemulsion.

**Table 9:** Hematological parameters analysis after 14 days of treatment with free chalcones.

Parameters	Control	R7	R13	R15
Total RBC count (×10^5^/mm^3^)	53 ± 4	57 ± 2	57 ± 2	59 ± 2
Leucocytes (×10^2^/mm^3^)	59 ± 13	46 ± 5	53 ± 9	59 ± 10
MCV (fL)	87 ± 1	87 ± 4	85 ± 2	86 ± 2
HCM (pg)	28 ± 1	26 ± 3	24 ± 2	27 ± 2
MCHC (%)	33 ± 1	30 ± 4	28 ± 2	31 ± 3
Hemoglobin (g/dL)	15 ± 1	15 ± 2	14 ± 3	16 ± 4
Hematocrit (%)	46 ± 4	50 ± 3	49 ± 2	51 ± 4
Neutrophils (×10^2^/mm^3^)	11 ± 3	14 ± 2	17 ± 4	22 ± 3
Band cells (×10^1^/mm^3^)	11 ± 3	18 ± 9	16 ± 11	24 ± 12
Mononuclear (×10^2^/mm^3^)	47 ± 11	30 ± 2	32 ± 4	36 ± 5
Eosinophils (×10^1^/mm^3^)	-	-	-	-
Basophils (×10^1^/mm^3^)	-	14 ± 5	27 ± 10 ^*^	-

* *p* ≤ 0.05 using ANOVA followed by Dunnet’s test when compared with the control group.

**Table 10:** Hematological parameters analysis after 14 days of treatment with chalcone-loaded nanoemulsions.

Parameters	Control	NE	NE/R7	NE/R13	NE/R15
Total RBC count (×10^5^/mm^3^)	53 ± 4	68 ± 4	45 ± 2	54 ± 7	57 ± 4
Leucocytes (×10^2^/mm^3^)	59 ± 13	91 ± 3 ^**^	85 ± 2 ^*^	66 ± 2	64 ± 3
MCV (fL)	87 ± 1	81 ± 8	92 ± 7	88 ± 4	85 ± 3
HCM (pg)	28 ± 1	26 ± 4	33 ± 4	27 ± 3	26 ± 5
MCHC (%)	33 ± 1	32 ± 3	33 ± 2	31 ± 4	31 ± 2
Hemoglobin (g/dL)	15 ± 1	18 ± 3	15 ± 3	15 ± 2	15 ± 1
Hematocrit (%)	46 ± 4	55 ± 5	45 ± 2	48 ± 4	48 ± 2
Neutrophils (×10^2^/mm^3^)	11 ± 3	21 ± 2 ^**^	10 ± 2	10 ± 1	10 ± 1
Band cells (×10^1^/mm^3^)	11 ± 3	9 ± 9	25 ± 8	20 ± 13	6 ± 6
Mononuclear (×10^2^/mm^3^)	47 ± 11	69 ± 4 ^*^	72 ± 3 ^*^	50 ± 3	54 ± 2
Eosinophils (×10^1^/mm^3^)	-	9 ± 9	-	-	-
Basophils (×10^1^/mm^3^)	-	9 ± 9	17 ± 17	26 ± 13	-

* *p* ≤ 0.05 and ** *p* ≤ 0.01 using ANOVA followed by Dunnet’s test when compared with the control group.

### 3.6. Oxidative Stress Evaluation

The results obtained for all parameters of oxidative stress evaluated in the liver homogenates are shown in Table 11 and Table 12. All chalcones induced an increase in the amount of reactive oxygen species (ROS) and lipid peroxidation (TBARS), followed by a decrease in GSH, characterizing an oxidative stress. Encapsulation of R7 completely avoided the oxidative stress. Contrariwise, the encapsulation of R13 did not change any evaluated parameter. Encapsulation of R15 avoided the increase in ROS and lipid peroxidation, but did not increase the antioxidant defense (GSH).

**Table 11:** Liver oxidative stress analysis after 14 days of treatment with free chalcones.

Parameters	R7 (%)	R13 (%)	R15 (%)
TBARS	213 ± 22 ^***^	148 ± 18	139 ± 4
ROS	158 ± 11 ^*^	181 ± 6 ^**^	228 ± 20 ^**^
GSH	57 ± 8 ^**^	29 ± 3 ^***^	45 ± 5 ^***^
Catalase	160 ± 23	135 ± 11	130 ± 16
GR	92 ± 10	103 ± 7	79 ± 8
GPx	96 ± 3	101 ± 1	80 ± 9
GST	99 ± 12	108 ± 9	81 ± 10

* *p* ≤ 0.05; ** *p* < 0.01; *** *p* < 0.001 using ANOVA followed by Dunnet’s test when compared with the control group. Results are expressed in relation to the control considered 100%. Controls values: 21 nmoL/mg protein for TBARS; 12 µmoL/mg protein for GSH; 12 µmoL/min/µg protein for catalase; 1 µmoL/min/µg protein for GR; 14 µmoL/min/µg protein for GPx and GST.

**Table 12:** Liver oxidative stress analysis after 14 days treatment with chalcones-loaded nanoemulsions.

Parameters	NE (%)	NE/R7 (%)	NE/R13 (%)	NE/15 (%)
TBARS	144 ± 10 ^**^	109 ± 3	125 ± 5	109 ± 7
ROS	95 ± 14	129 ± 8	147 ± 17	107 ± 10
GSH	118 ± 6	100 ± 3	46 ± 7 ^**^	46 ± 7 ^**^
Catalase	84 ± 4	61 ± 9 ^*^	31 ± 8 ^**^	77 ± 5
GR	106 ± 4	111 ± 6	102 ± 14	109 ± 21
GPx	89 ± 3	100 ± 3	95 ± 4	103 ± 4
GST	113 ± 14	99 ± 13	101 ± 15	113 ± 9

* *p* ≤ 0.05; ** *p* < 0.01 using ANOVA followed by Dunnet’s test when compared with the control group. Results are expressed in relation to the control considered 100%. Controls values: 21 nmoL/mg protein for TBARS; 12 µmoL/mg protein for GSH; 12 µmoL/min/µg protein for catalase; 1 µmoL/min/µg protein for GR; 14 µmoL/min/µg protein for GPx and GST.

### 3.7. Histopathological Analysis

**Figure 2:**
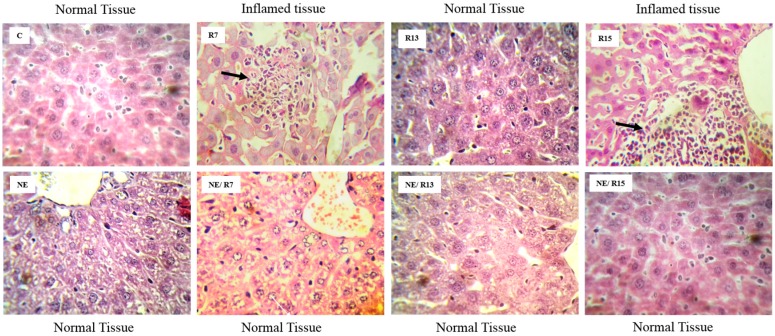
Histological analysis of livers after 14 days with free and chalcones-loaded nanoemulsions. Black arrows indicate inflammatory cells.

Histopathological analysis revealed inflammatory cell infiltration in livers of the animals treated with R7 and R15. The treatment with chalcone-loaded nanoemulsion circumvented this process (Figure 2). The chalcone R13-treatment did not induce any liver injury.

## 4. Discussion

Because of their biocompatibility, lipid nanoparticles (LPs) have been widely used in the medical field for delivery and controlled release of anticancer drugs. Among LPs, nanoemulsions have started evolving as carriers for the delivery of hydrophobic drugs, because they present a very large surface area and good long-term stability [13,37]. In chemotherapy, drug-loaded nanoparticles have been used to avoid common problems such as poor specificity, high toxicity to non-tumoral cells and susceptibility to the induction of drug resistance [11]. The utilization of nanoparticles as drug delivery agents in leukemia has several advantages, including specific targeting via receptor-mediated mechanisms. Many works have demonstrated the importance of lipoprotein and folate receptors in the uptake of nanoparticles loaded with different drugs [38,39,40,41]. Satake and collaborators developed a nanoparticle with a peptidomimetic ligand (LLP2A) targeted against an activated α4β1 integrin, which is highly expressed in childhood ALL [42]. CD22 is a receptor B-cell-specific and have been studied as a novel approach for targeting B-cell leukemias. Several antibody-loaded nanoparticles therapies targeting CD22 are being studied because CD22 is an endocytic receptor and nanoparticles are rapidly internalized by the cell [43,44].

The chalcones studied in this work are known to present good antileukemic activity, inducing cell death by different apoptosis pathways [15,16], but also cytotoxicity to non-tumoral cells. Thus, we prepared chalcone-loaded nanoemulsions and compared their antileukemic activity *in vitro*, and toxic effects *in vivo*, with the respective effects of the free compounds. 

Despite the relative selectivity of nanoemulsions for leukemic cells, the cytotoxicity of chalcones was similar when they were in bulk solution or in a nanoemulsion-loaded form. The lack of increase of* in vitro* anti-tumoral effect using encapsulated drugs was also observed in other studies [18,45,46,47] and can be related with the limitations of* in vitro* studies. To better compare the antitumoral effects of free chalcones and nanoemulsion-loaded ones, an* in vivo* study using a tumor model needs be conducted. As indicated in other studies performed* in vivo*, encapsulated drugs normally present a higher antitumoral effect than the free compounds [45,48,49]. More specifically for leukemia, the encapsulation could increase the permeation of drugs through the blood brain barrier, which is the extra-medullar organ where a high incidence of metastasis occurs [50,51,52]. 

The results from* in vitro* assays obtained in this study demonstrated that the encapsulation decreased the cytotoxicity of chalcones in non-tumoral cells and maintained the cytotoxicity in leukemic cell lines. The higher selectivity of chalcone-loaded nanoemulsions for leukemic cells is thought to be a consequence of the higher uptake of nanoemulsions by these cells compared with non-tumoral ones. Leukemic cells need much more energy for proliferation than non-tumoral cells and therefore present higher LDL-receptor expression [53,54]. The increase of LDL receptors in patients with acute leukemia can be 3 to 100 times higher than in healthy patients [55]. Nanoemulsions present a similar composition to LDL and could therefore accumulate more easily in leukemic cells than in non-tumoral cells. Similar works have demonstrated a high selectivity of lipid nanoparticles for tumoral cells [56,57,58,59,60]. 

In order to better characterize the toxicity of chalcones,* in vivo* studies with healthy mice were performed, and the results demonstrated that the encapsulation decreased the toxicity of chalcones as observed in the results obtained* in vitro* with VERO cells. The general toxicity of free chalcones was evidenced by a reduction of weight gain of animals, an effect that was circumvented by chalcone encapsulation. 

Blank nanoemulsion (NE) induced an increase in leucocytes and neutrophils cells. Nanoparticles might activate the immune system and thus affect the therapeutic efficacy, as in the case of cancer treatment and vaccination. Conversely, an undesirable immune-stimulation might induce cytokine storm, interferon response and/or lymphocytes activation causing severe adverse effects [61].

Free chalcones also seem to induce liver injury, evidenced by diffuse cloudy hepatocellular swelling and vacuolated cytoplasms, as well as inflammatory infiltration predominantly in the periportal area. Consistent with histological data, free chalcones also induced an increase in ROS and lipid peroxidation, and a decrease in GSH in the animal’s liver. The cell death mechanism of chalcones R7, R13 and R15 in a leukemic cell line is associated with oxidative stress [15]. The liver injuries induced in animals, observed here, could also be a result of an oxidative stress process. Furthermore, the liver is the organ responsible for detoxification of drugs and metabolites, and many injuries in this organ are related to drugs such as acetaminophen, or to all anticancer drugs used in leukemia treatment [62,63]. Despite the liver injury caused by the chalcones, it seems to be reversible and well tolerated by the animals because no death or behavioral changes were observed. 

Our results also show that the encapsulation of chalcones R7 and R15 impaired ROS generation and lipid peroxidation, and chalcone R7 avoided the decrease of GSH in liver. Both chalcones also impaired the inflammatory process observed in histological evaluation. All results point to the conclusion that encapsulation reduces liver injury induced by free chalcones. 

## 5. Conclusions

Given the fact that most anticancer drugs present serious side effects, research into new drugs is needed, as well as is the search for new technologies to improve therapies and patients’ life quality. In this work, it has been shown that nanoemulsions maintained the antileukemic effect of chalcones and decreased their toxic effect in non-tumoral cells and in animals. Although not completely innocuous, nanoemulsions provide an interesting biocompatible strategy to decrease drug-induced side effects without losing antitumoral efficacy.

## Supplementary Files

Supplementary File 1
