# Supplementary material for: In vitro and in vivo Effects of Free and Chalcones-Loaded Nanoemulsions: Insights and Challenges in Targeted Cancer Chemotherapies
Source: Int J Environ Res Public Health. 2014 Sep 26;11(10):10016–35. doi: 10.3390/ijerph111010016 (PMC4210964; doi:10.3390/ijerph111010016)
Supplement: Supplementary File 1 [file ijerph-11-10016-s001.pdf]

Supplementary Information

In vitro and *in vivo* Effects of Free and Chalcones-Loaded Nanoemulsions: Insights and Challenges in Targeted Cancer Chemotherapies

---

Figure S1. Heating curves of differential scanning calorimetry (DSC) for free chalcone (solid line), lyophilized chalcones-loaded nanoemulsions (dash line) and blank nanoemulsion (dot line). (**A**) R7; (**B**) R13; (**C**) R15.

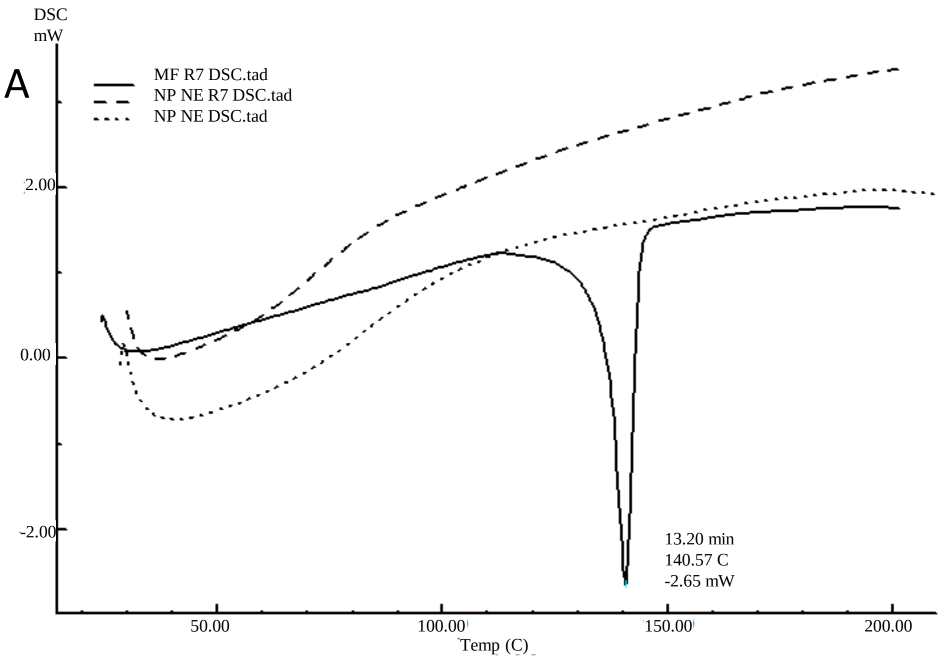

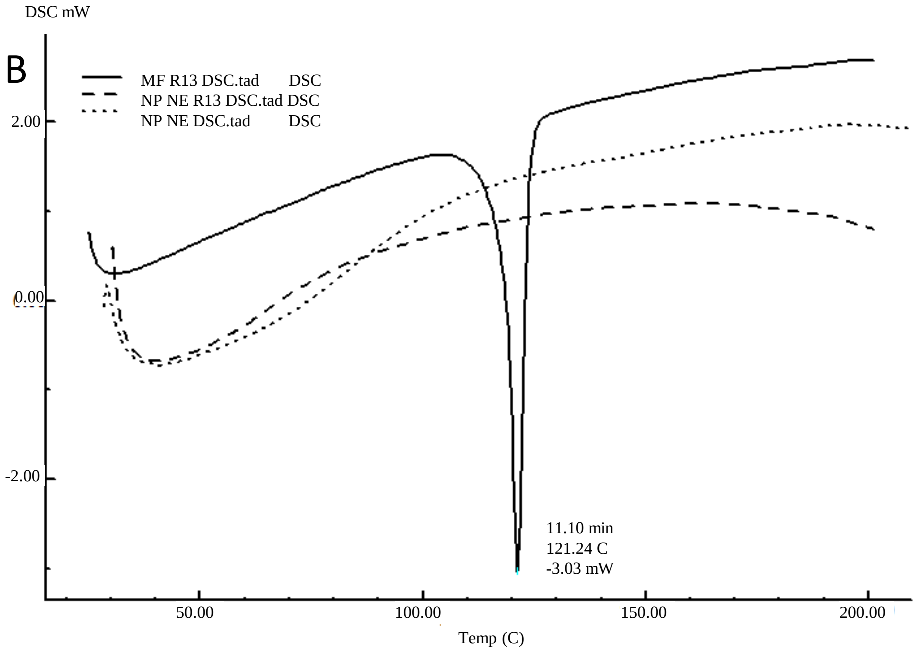

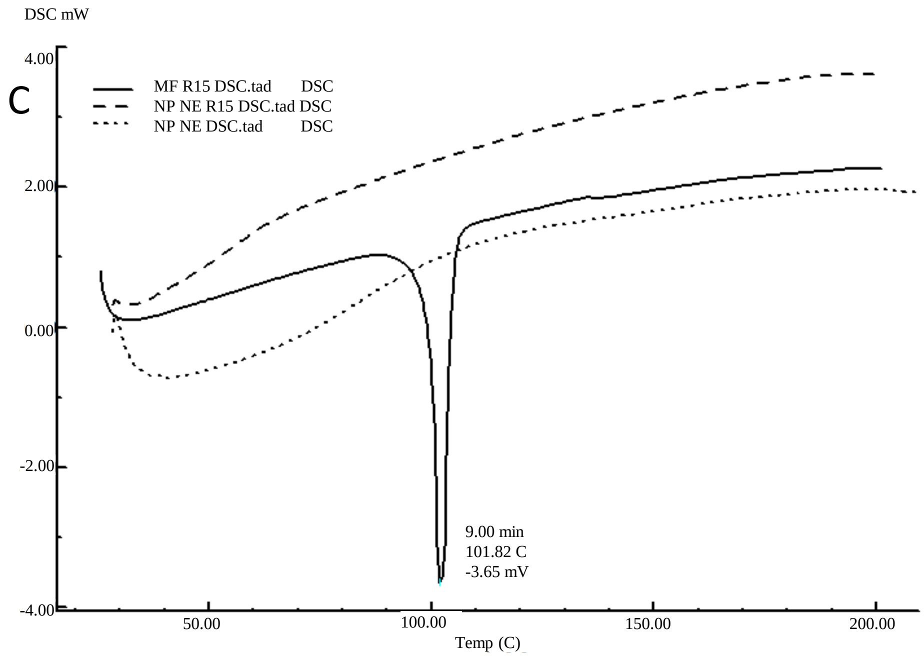

© 2014 by the authors; licensee MDPI, Basel, Switzerland. This article is an open access article distributed under the terms and conditions of the Creative Commons Attribution license (http://creativecommons.org/licenses/by/4.0/).
